# Psoriasis: rare case of erythrodermic psoriasis on the lower extremity

**DOI:** 10.11604/pamj.2025.52.25.48328

**Published:** 2025-09-18

**Authors:** Sharayu Bhaskarrao Balkhande

**Affiliations:** 1 Department of Rachana Sharir, Mahatma Gandhi Ayurved College Hospital and Research Centre, Datta Meghe Institute of Higher Education and Research (Deemed to be University), Sawangi, Wardha, Maharashtra, India

**Keywords:** Erythrodermic psoriasis, Erythema, scaling

## Image in medicine

Erythrodermic psoriasis is a rare and severe form of psoriasis characterised by widespread erythema (redness), scaling, and inflammation that covers most of the body. It is considered a medical emergency due to its potential to cause significant systemic complications, including fluid and electrolyte imbalances and infections. A 45-year-old patient with a known history of psoriasis presents with severe erythrodermic psoriasis confined to both legs. Over the past few years, the patient has developed extensive erythema, thick scaling, and inflamed patches on both legs, with the lesions merging into large, confluent plaques. The skin appears red, swollen, and tender, with noticeable dryness and fissuring in some areas (A,B,C). The patient reported significant discomfort, including burning sensations and intense itching. Systemic symptoms such as fever, chills, and fatigue were present; the patient denied any recent infections or major medication changes but attributed the flare-up to increased emotional stress. Given the severity of the presentation and the risk of complications, treatment was initiated with high-potency corticosteroids to reduce inflammation and vitamin D analogues to normalise skin cell turnover. Systemic therapies, such as methotrexate or biologics, were considered for more aggressive control, and close monitoring for signs of dehydration, electrolyte imbalances, or secondary infections was essential. The patient would require an ongoing follow-up to adjust treatment and manage potential relapses.

**Figure 1:**
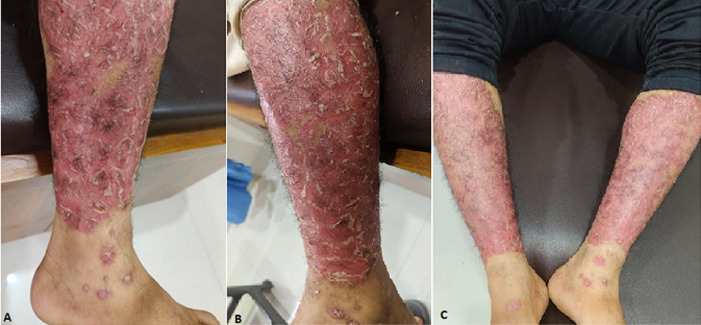
A) erythrodermic psoriasis on right leg; B) erythrodermic psoriasis on left leg; C) erythrodermic psoriasis over both

